# International initiative for a curated *SDHB* variant database improving the diagnosis of hereditary paraganglioma and pheochromocytoma

**DOI:** 10.1136/jmedgenet-2020-107652

**Published:** 2021-08-27

**Authors:** Laurene Ben Aim, Eamonn R Maher, Alberto Cascon, Anne Barlier, Sophie Giraud, Tonino Ercolino, Pascal Pigny, Roderick J Clifton-Bligh, Delphine Mirebeau-Prunier, Amira Mohamed, Judith Favier, Anne-Paule Gimenez-Roqueplo, Francesca Schiavi, Rodrigo A Toledo, Patricia L Dahia, Mercedes Robledo, Jean Pierre Bayley, Nelly Burnichon

**Affiliations:** 1 Genetics Department, Assistance Publique-Hôpitaux de Paris (AP-HP), Hôpital Européen Georges Pompidou, Paris, France; 2 Department of Medical Genetics, University of Cambridge and NIHR Cambridge Biomedical Research Centre, Cambridge, UK; 3 Hereditary Endocrine Cancer Group, CNIO, Madrid, Spain; 4 Laboratory of Molecular Biology, La Conception Hospital, Marseille, France; 5 Department of Genetics, Hospices Civils de Lyon, Bron, France; 6 Endocrinology Unit, Azienda Ospedaliero-Universitaria Careggi, Firenze, Italy; 7 Institut de Biochimie & Biologie Moléculaire, Lille University Hospital Center, Lille, France; 8 Department of Endocrinology, Royal North Shore Hospital, Kolling Institute, University of Sydney, Sydney, New South Wales, Australia; 9 Département de Biochimie et Génétique, CHU Angers, Angers, France; 10 Université de Paris, PARCC, INSERM, Equipe Labellisée par la Ligue contre le Cancer, Paris, France; 11 Familial Cancer Clinic and Oncoendocrinology, IOV IRCCS, Padova, Italy; 12 CIBERONC, Gastrointestinal and Endocrine Tumors, VHIO, Barcelona, Spain; 13 Department of Medicine, Division of Hematology and Medical Oncology, Mays Cancer Center, University of Texas Health Science Center at San Antonio, San Antonio, Texas, USA; 14 Human Genetics, LUMC, Leiden, The Netherlands

**Keywords:** genetic variation, databases, genetic, adrenal gland diseases, genetic testing, human genetics

## Abstract

**Background:**

*SDHB* is one of the major genes predisposing to paraganglioma/pheochromocytoma (PPGL). Identifying pathogenic *SDHB* variants in patients with PPGL is essential to the management of patients and relatives due to the increased risk of recurrences, metastases and the emergence of non-PPGL tumours. In this context, the ‘NGS and PPGL (NGSnPPGL) Study Group’ initiated an international effort to collect, annotate and classify *SDHB* variants and to provide an accurate, expert-curated and freely available *SDHB* variant database.

**Methods:**

A total of 223 distinct *SDHB* variants from 737 patients were collected worldwide. Using multiple criteria, each variant was first classified according to a 5-tier grouping based on American College of Medical Genetics and NGSnPPGL standardised recommendations and was then manually reviewed by a panel of experts in the field.

**Results:**

This multistep process resulted in 23 benign/likely benign, 149 pathogenic/likely pathogenic variants and 51 variants of unknown significance (VUS). Expert curation reduced by half the number of variants initially classified as VUS. Variant classifications are publicly accessible via the Leiden Open Variation Database system (https://databases.lovd.nl/shared/genes/SDHB).

**Conclusion:**

This international initiative by a panel of experts allowed us to establish a consensus classification for 223 *SDHB* variants that should be used as a routine tool by geneticists in charge of PPGL laboratory diagnosis. This accurate classification of *SDHB* genetic variants will help to clarify the diagnosis of hereditary PPGL and to improve the clinical care of patients and relatives with PPGL.

## Introduction

The SDHB protein (OMIM185470) corresponds to the catalytic iron-sulfur subunit of the heterotetrameric succinate dehydrogenase (SDH) complex, a component of both the tricarboxylic acid cycle and the mitochondrial respiratory chain (complex II). Germline inactivating *SDHB* pathogenic variants were first identified in kindreds with familial pheochromocytoma and paraganglioma (PPGL).^1^ Paragangliomas (PGL) and pheochromocytomas (PCC) are rare neuroendocrine tumours arising from the sympathetic/parasympathetic ganglia and the adrenal medulla, respectively. Among human tumours, PPGLs show the highest frequency of hereditary forms of the disease, representing at least 35% of all cases.^2^ For this reason, some PPGL guidelines recommend that genetic testing should be considered in all patients with PPGL.^3 4^ Over 20 genes are known to increase susceptibility to PPGL. *SDHB*, which acts as a tumour suppressor gene, is one of the most frequently involved, considering that around 10% of all PPGL cases carry an *SDHB*-pathogenic variant, including patients with apparently sporadic disease.^2 5–7^ *SDHB* therefore is included in the primary (also referred to as ‘basic’) panel of genes routinely analysed in patients with PPGL, all of which were validated as bona fide susceptibility genes.^2 4^ *SDHB* pathogenic variants most commonly predispose to head and neck PGLs, sympathetic PGLs and PCCs, and also give rise to renal cell carcinomas (RCC), wild-type gastrointestinal stromal tumours (wtGIST) and pituitary adenomas.^8^ Moreover, *SDHB* pathogenic variants have been associated with a high risk of metastasis and a poor prognosis.^9 10^ As *SDHB* patients face a high risk of disease recurrence, possible emergence of non-PPGL tumours such as RCCs and wtGISTs and metastatic evolution, it is essential to patient management and follow-up that the identification of an *SDHB* variant is followed by an accurate classification as ‘disease causing’ or not. Determining the pathogenicity of an *SDHB* variant is also a precondition for presymptomatic genetic testing of relatives and the appropriate management of carriers of pathogenic variants. Indeed, the ‘unclassified genetic variants’ working group at the International Agency for Research on Cancer recommends that predictive genetic testing of at-risk relatives should be confined to pathogenic or likely pathogenic variants.^11^

Public gene-specific databases already exist for various PPGL susceptibility genes. The NGS in PPGL (NGSnPPGL) Study Group—which includes 18 experts from 8 countries and includes both clinicians and basic researchers involved in PPGL genetic testing and genetic counseling—published a consensus statement in 2017 emphasising the importance of multi-institutional, internationally shared efforts to compile resources for genomic, clinical and functional data, including the comprehensive annotation of variants and encouraging public sharing of novel variants.^2^

The *SDHB* variant database was created in 2005, using the Leiden Open Variation Database (LOVD) platform (https://databases.lovd.nl/shared/genes/SDHB).^12^ The database mainly consists of variants extracted from the published literature but increasingly includes directly submitted variants. As of September 2020, the LOVD-*SDHB* comprises 288 unique variants for which a detailed nomenclature, clinical and segregation data, as well as publication references are available.^13^

Here, we present an international initiative of the NGSnPPGL study group to provide a comprehensive annotation of *SDHB* variants, to gather variants identified in laboratories worldwide that perform *SDHB* molecular analysis and to collect extensive data associated with these variants including patient phenotype and in silico, functional or additional analyses performed in each case. Based on these data, each variant was classified according to both the American College of Medical Genetics and Genomics (ACMG) and the Association for Molecular Pathology criteria,^14^ and the NGSnPPGL guidelines,^2^ into one of five classes of pathogenicity.^11^ All variants were then manually reviewed by a panel of international experts in the field, leading to a consensus classification. Our main goal was to provide a freely available, expert-curated, *SDHB* variant database, offering an accurate and harmonised interpretation that will improve the care of patients and families carrying these variants.

## Methods

### Data collection

Data on patients carrying an *SDHB* variant were submitted by 10 laboratories worldwide, which routinely perform the *SDHB* genotyping and belong to the European Network for the Study of Adrenal Tumors (ENS@T) and/or the Pheochromocytoma and paraganglioma RESearch Support Organization (PRESSOR) and/or the French Oncogenetics Network of Rare Neuroendocrine Tumors (TENgen network). Data on 737 index cases were collected, including 386 from France (5 laboratories located in Georges Pompidou European Hospital—Paris, La Conception hospital—Marseille, Hospices Civils—Lyon, Centre hospitalo-universitaire—Lille and Centre hospitalo-universitaire—Angers), 215 from the UK (Cambridge/Birmingham), 71 from Spain (Madrid), 32 from the USA (San Antonio, Texas), 20 from Italy (Firenze) and 13 from Australia (Sydney). Patients carrying only well-known single nucleotide polymorphisms of *SDHB* gene were not enrolled.

Clinical data collected for each index case included the type, number and site of tumours; the age at diagnosis; the metastatic status (defined by the presence of metastases in non-paraganglial tissue^15^); the biochemical secretion profile and the family history of *SDHB* spectrum tumours. Co-segregation data in families were reported when available.

All data have been collected retrospectively and de-identified.

### Molecular data

The nuclear *SDHB* gene (1p36) covers 8 exons and encodes a 281 amino acid protein.

Genotyping of leucocyte DNA was performed locally and included the sequencing of the full coding regions and exon-intron junctions using the Sanger or the next-generation sequencing (NGS) methods together with search for large rearrangements using Multiplex Ligation-dependent Probe Amplification (MLPA, MRC-Holland).

All variants were annotated according to the NM_003000.2 reference transcript on genome assembly GRCh37 (hg19) and following Human Genome Variation Society nomenclature (https://varnomen.hgvs.org/). For each variant, the DNA and protein changes were collected. Other tested genes and the co-occurrence of additional variants of interest were specified by submitters when pertinent.

Previous reports for each variant were checked in dbSNP, exome variant server, gnomAD and ClinVar databases through AlamutVisual software, V.2.10 (Interactive Biosoftware, Sophia Genetics) (online supplemental table 1). Variant frequencies in reference populations and literature references were included when available.

10.1136/jmedgenet-2020-107652.supp1 Supplementary data

### In silico predictions

For missense variants, in silico analyses were performed based on amino acid conservation among 14 orthologs and expressed as a ratio between 0 and 1 (*x* orthologs sharing the same amino acid/14), nucleotide conservation was based on PhyloP and Grantham distance score and predicted impact were derived from PolyPhen2 (HumVar), SIFT, Align-GVGD and Mutation Taster. Predicted impact according to MaxEntScan and NNSplice was estimated for variants with a potential splice effect (splicesite synonymous and intronic variants). The tools used and the corresponding thresholds are described in online supplemental table 1.

### Additional data

Supporting information such as loss of heterozygosity (LOH) in tumour DNA, cDNA analysis, SDHB and/or SDHA immunohistochemistry on FFPE tumour tissue sections, SDH enzymatic activity and other pertinent assays were reported when available (online supplemental table 2). Because *SDHB* functions as a tumour suppressor gene with loss of the wild-type allele in tumour tissue, LOH analysis was performed when both germline and tumour samples were available. In case of variants possibly leading to abnormal splicing, cDNA analysis was performed when leucocyte or tumour RNA was available. SDHB immunohistochemistry and SDH enzymatic activity measurement are useful tools to discriminate *SDHx*-related and non-*SDHx-*related tumours, as a loss of SDHB protein or SDH activity reliably signals the presence of an *SDHx* deleterious variant in tumour cells.^16 17^ Negative SDHB immunostaining or loss of SDH activity, combined with negative *SDHC*, *SDHD* and *SDHA* (or positive SDHA immunohistochemistry^18^) genotyping, is a hallmark of *SDHB* loss-of-function variants. When available, tumour classification in cluster 1A (*SDHx*-related), 1B (*VHL*-related) or 2A (*RET*/*NF1*/sporadic tumours), according to previous transcriptomic analyses^19 20^ was specified, as well as SDHB protein expression as evaluated by western blot analysis (online supplemental figure 1).

10.1136/jmedgenet-2020-107652.supp2 Supplementary data

10.1136/jmedgenet-2020-107652.supp3 Supplementary data

### Variant classifications

All variants were classified according to ACMG criteria^14^ adjusted to *SDHB* gene and PPGL specificities (online supplemental figure 1). As they are not applicable and/or established for the *SDHB* gene, the following criteria were not considered: PS4 (prevalence in affected individuals statistically increased over controls); BP1 (missense in gene where only truncating cause disease); PP2 (missense in gene with low rate of benign missense variants and pathogenic missenses common); PM1 (mutational hot spot or well-studied functional domain without benign variation); PM3 (for recessive disorders, detected in trans with a pathogenic variant); PM6 and PS2 (because de novo cases are very rare, *SDHB* variants are usually inherited, even from unaffected parents due to low penetrance of the disease). As there are multiple PPGL susceptibility genes, PP4 criteria (patient’s phenotype or family history highly specific for gene) were not retained for classification. Additional data criteria were adapted as follows: benign strong (loss of the allele carrying the *SDHB* variant in tumour DNA), benign supporting (no LOH at the *SDHB* locus, assay not in favour of *SDHB* loss of function), pathogenic supporting (LOH at the *SDHB* locus), pathogenic moderate (assay in favour of SDH loss of function without all *SDHx* genes analysed), pathogenic strong (evidence for SDH loss of function and no additional variant found in *SDHx* genes), pathogenic very strong (evidence for SDHB-specific loss of function) (online supplemental figure 1). We also considered other criteria calibrated between benign strong and pathogenic strong according to number of published cases and previously reported evidence.

Variants were also classified according to the NGSnPPGL study group framework^2^ based on the frequency of the variant in the general population, the variant type, in silico predictions, co-segregation in families and the results of additional information or assays available in the literature or for the submitted cases (online supplemental figure 2).

10.1136/jmedgenet-2020-107652.supp4 Supplementary data

Both classifications assigned each variant to one of the following classes: benign variant (BV, class 1), likely benign variant (LBV, class 2), variant of unknown significance (VUS, class 3), likely pathogenic variant (LPV, class 4) or pathogenic variant (PV, class 5).

After data collection, in silico analyses and variant classification according to both ACMG and NGSnPPGL study group guidelines were performed by a sole investigator (LBA) for consistency.

### Curation by experts

After standardised ACMG and NGGnPPGL study group classification, manual review of each variant was independently carried out by five experts (NB, JPB, MR, FS, AC), which involved choosing between two assigned classes when they were different, or applying a different classification based on personal expertise.

In case of discordance between expert classifications after the first round of reviewing, a second round of expert curation (NB, JPB, MR, PLD, RAT) was performed in order to achieve a consensus class for each variant.

## Results

### Characterisation of the patient cohort

Clinical data were collected for 737 index cases carrying a *SDHB* variant (table 1).

**Table 1:** Clinical presentation of patients included in the study

Clinical data		All index cases with *SDHB* variant (n=737)	Index cases with (likely) pathogenic *SDHB* variant (n=614)
Number and location of tumours	Single PPGL	618 (84.5%)	514 (84.3%)
	Single headandneck PGL	213 (44.8%)	171 (44.5%)
	Single TAP-PGL	184 (38.7%)	166 (43.2%)
	Single PCC	78 (16.4%)	47 (12.2%)
	Location not specified	*143*	*130*
	Multiple PPGL	103 (14.1%)	89 (14.6%)
	RCC only	8 (1.1%)	5 (0.8%)
	GIST only	2 (0.3%)	2 (0.3%)
Number and location of tumours not specified		*6*	*4*
Benign/metastatic status	Metastatic disease	133 (35.8%)	122 (40%)
	Benign disease	238 (64.2%)	183 (60%)
Metastatic status not specified		*366*	*309*
Familial/Sporadic presentation	Family history	159 (29.8%)	142 (33.1%)
	No family history	374 (70.2%)	287 (66.9%)
Family history not specified		*204*	*185*

GIST, gastrointestinal stromal tumour; PCC, pheochromocytoma; PGL, paraganglioma; PPGL, paraganglioma/pheochromocytoma; RCC, renal cell carcinoma; TAP-PGL, thoracic, abdominal or pelvic PGL.

Patients were affected by a single PPGL in 84.5% (618/731) of cases, multiple (2–7) PPGL in 14.1% (103/731), RCC only in 1.1% (8/731) and GIST only in 2 cases. The tumour location was unknown for six patients. Among patients affected by a single PPGL with known location, 213 had a head and neck PGL (44.8%), 184 a thoracic, abdominal or pelvic PGL (38.7%) and 78 a PCC (16.4%). The precise tumour location was not specified for other patients (n=143). Metastatic disease was diagnosed in 133 patients, corresponding to 35.8% of cases with known benign/metastatic status. When family history was known, the disease was familial in 29.8% of cases (159/533). Twenty-three per cent of patients were affected during their fourth decade. The minimum age at diagnosis was 6 years, the mean age was 36 years and the maximum age was 83 years.

### Variant characterisation

**Figure 1:**
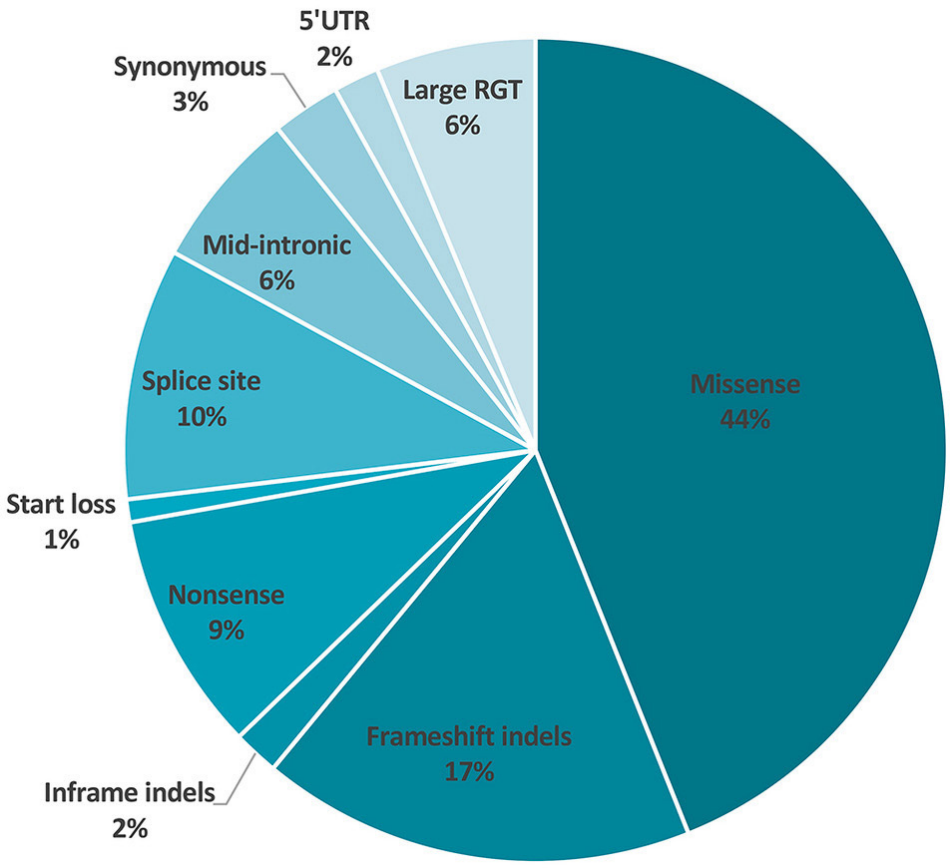
Distribution of *SDHB* variants according to the type of alteration RGT: rearrangement. UTR, untranslated region.

**Figure 2:**
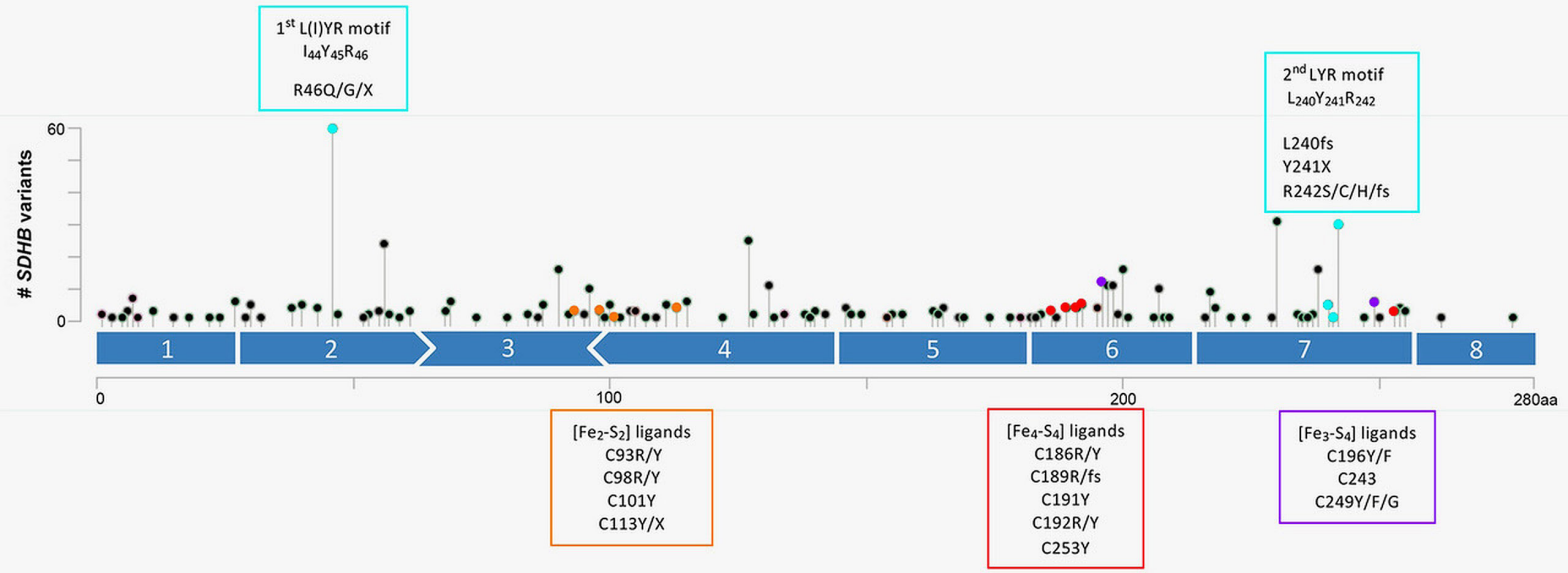
Diagram of the coding *SDHB* variants along the amino acid sequence L(I)YR motifs are shown in blue. The cysteine residues ligating the [Fe_2_-S_2_], the [Fe_4_-S_4_] and the [Fe_3_-S_4_] are shown in orange, red and purple, respectively. Diagram is displayed as lollipop symbols designed using the Mutation Mapper tool of the cBioPortal website. The Y axis represents the number of occurrences of variant in one residue.

A total of 223 distinct variants have been collected from the 737 entries (online supplemental table 2). Of these, 122 variants were found only once, 75 occurred 2–5 times, 12 were found 6–10 times, 9 variants occurred between 11 and 20 times and 5 variants were found 20 or more times. As regards the type of variant, 162 (73%) were nucleotide substitutions including missense (n=98, 44%), nonsense (n=21, 9%), splicesite (n=20, 9%), mid-intronic (n=12, 5%), synonymous (n=6, 3%), 5' untranslated region (n=3, 1%) and initiation codon (n=2, 1%) variants. Other variants consisted of 61 (27%) deletions or duplications including indels leading to a frameshift (n=38, 17%), large deletions/duplications (n=14, 6%), in-frame deletions (n=4, 2%) or indels affecting non-coding regions (n=3, 1%) or splicesites (n=2, 1%) (figure 1). *SDHB* exonic variants are distributed along the entire coding sequence of the gene (figure 2). Relative to exon size, *SDHB* substitutions or indels are more frequent in exon 7 (36 variants for 123 bp), exon 6 (29 variants for 102 bp) and exon 4 (36 variants for 137 bp). Exon 8 is rarely involved, with only two substitutions within 78 bp.

Of the 223 distinct variants, 166 had been previously described (135 in SDHB-LOVD, 107 in ClinVar, 84 in dbSNP, 49 in gnomAD and 14 in ESP). To our knowledge, 57 variants have not been previously reported in any database (online supplemental table 2).

### Variant classification based on ACMG and NGSnPPGL recommendations

**Figure 3:**
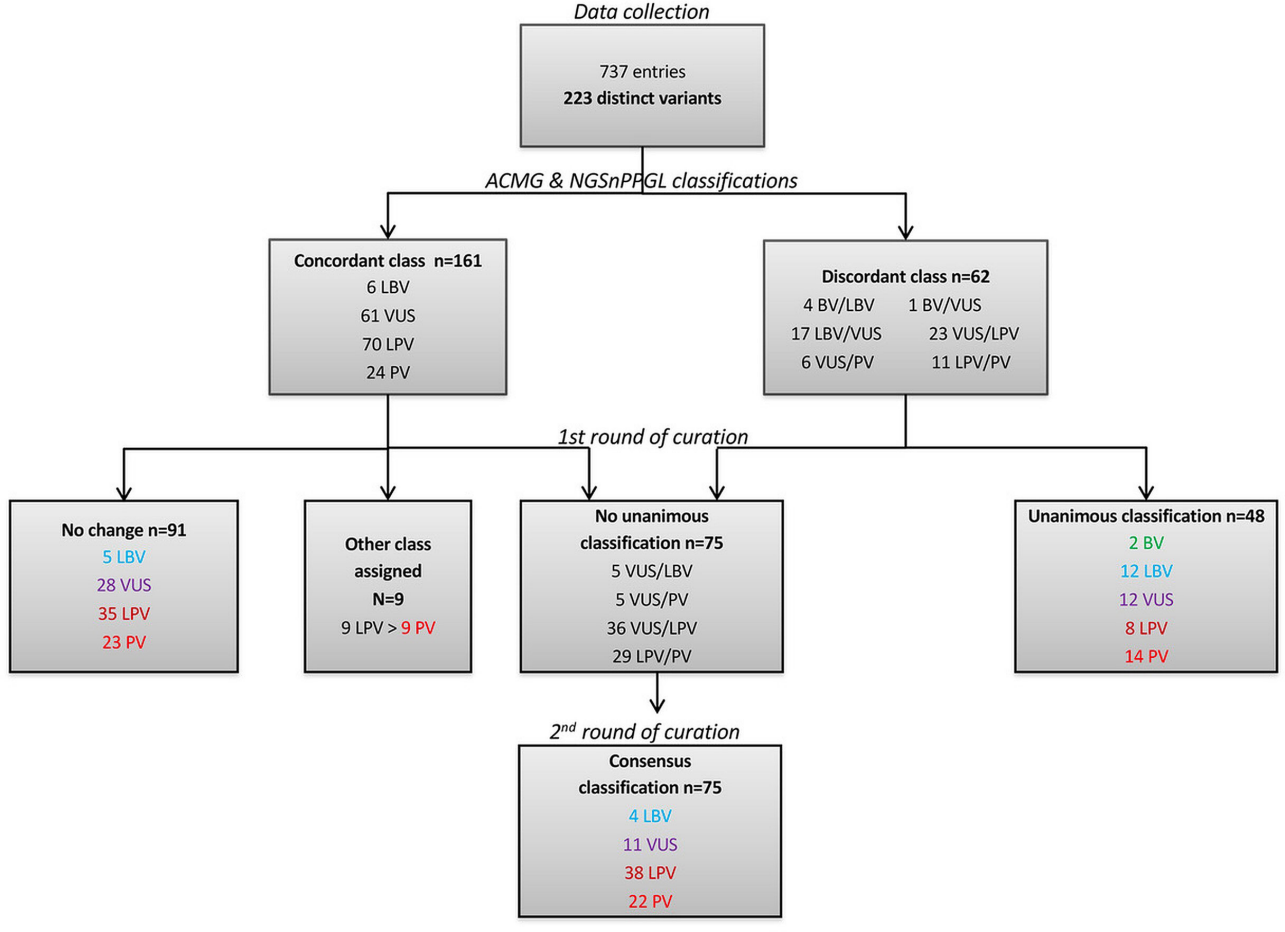
Strategy for variant classification. ACMG, American College of Medical Genetics and Genomics; BV, benign variant; LBV, likely benign variant; LPV, likely pathogenic variant; PV, pathogenic variant; VUS, variant of unknown significance.

Each variant was classified according to criteria from both the ACMG and NGSnPPGL frameworks (figure 3). This step led to concordant classification of 161 variants, comprising 6 LBV, 61 VUS, 70 LPV and 24 PV variants. Sixty-two variants showed discordant ratings based on ACMG and NGSnPPGL criteria, including 4 BV/LBV, 1 VUS/BV, 17 VUS/LBV, 23 LPV/VUS, 6 VUS/PV and 11 LPV/PV.

### Variant assessment by a panel of experts

After the first round of manual reviewing of all variants by the experts, the ACMG/NGSnPPGL classification, when concordant, remained unchanged for 91/161 variants (5 LBV, 28 VUS, 35 LPV and 23 PV). All experts agreed to convert nine originally LPV to PV including three gross deletions (deletion encompassing exons 1–7, exons 2–8 or the whole gene), two affecting splice junctions (c.423+1G>C and c.540G>C), 3 missense (p.Gly208Glu, p.Arg230His and p.Cys253Tyr) and one nonsense (p.Arg90Ter) variant, due to cumulative evidence of pathogenicity and/or high recurrence in patients.

For variants that were originally discordant between the two classification systems (n=62), the first round of curation led to the unanimous assignment of 48 variants to a single class, including 2 BV, 12 LBV, 12 VUS, 8 LPV and 14 PV (figure 3).

### Variant classification after the second round of curation

Following the first expert review, 75 variants remained without an unanimous classification, comprising 5 VUS or LBV, 5 VUS or PV, 36 VUS or LPV and 29 LPV or PV. These 75 variants were then submitted to a second round of expert review. Based on a careful review of additional data obtained during the reviewing process (new publications, SDHB immunohistochemistry results, description of new probands carrying the variant) and on their own expertise, the curators reached consensus on all variants.

**Figure 4:**
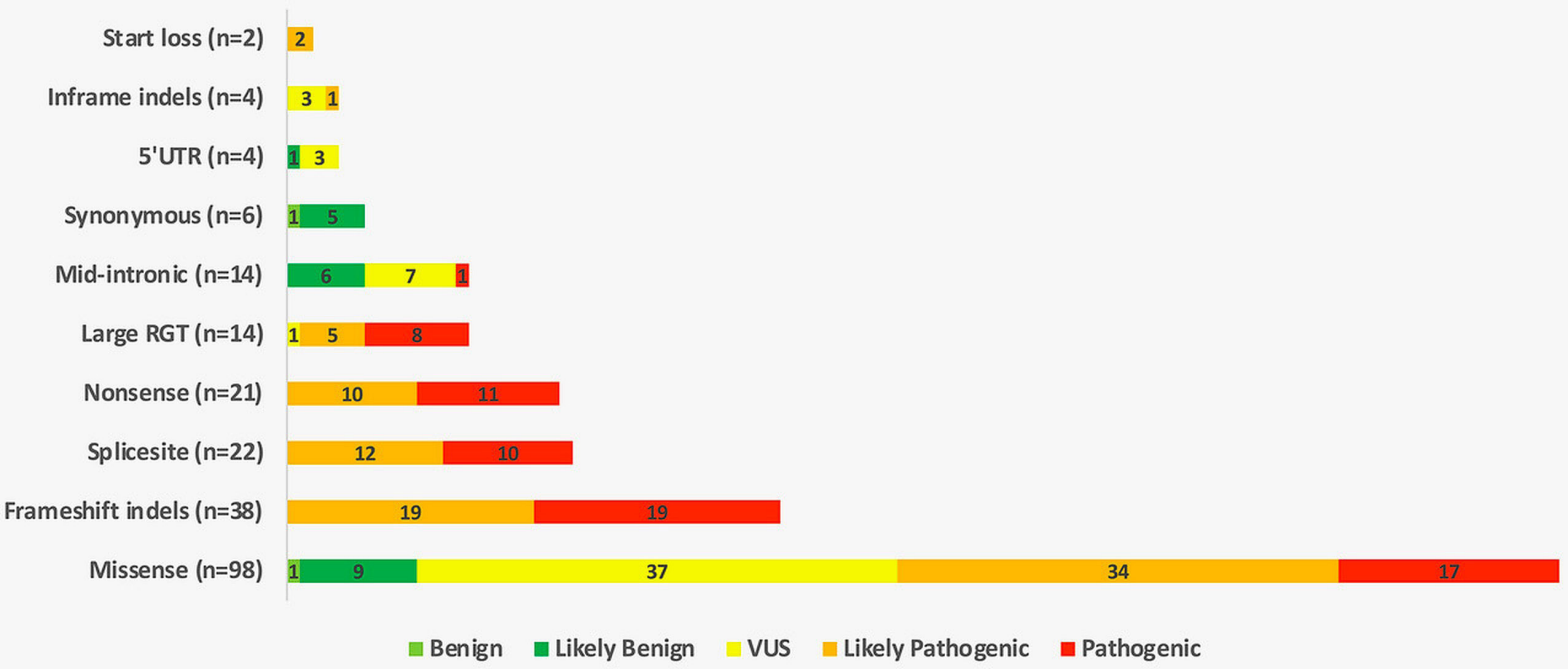
Distribution of *SDHB* variant types according to the 5-tier classification. VUS, variant of unknown significance; UTR, untranslated region.

After the final round of curation, the 223 distinct *SDHB* variants were classified as follows: 2 benign, 21 likely benign, 51 VUS, 83 likely pathogenic and 66 pathogenic variants (figures 3 and 4).

## Discussion

With extensive data collected from 737 *SDHB*-positive index cases, this study provides the first comprehensive overview of *SDHB* variants identified worldwide. The 223 distinct variants reported here were first classified according to a five-tier grouping using both the ACMG guidelines and the recommendations specifically designed for PPGL susceptibility genes established by the NGSnPPGL study group. All variants were then curated by a panel of international experts in the field based on two rounds of review. This multistep process resulted in a consensus classification for each variant. This expert-curated *SDHB* variant database is now freely available for the scientific community in the LOVD system (https://databaseslovdnl/shared/genes/SDHB).^12^

The distribution of variant types in this study is in accordance with prior reports.^21^ Regarding missense variants, 38% are located in either L(I)YR Fe-S transfer motifs or in the Fe-S cluster-ligating cysteines as previously reported (figure 2).^22^ The high rate of gross *SDHB* deletions or duplications, carried by 12% of index cases (86/737) and including hotspots such as exon 1 deletion (see below), is confirmed in this study and emphasises the importance of searching for large rearrangements using NGS or dedicated methods like MLPA.^23^ Most variants were reported only once (55%, n=123) but 14 were recorded >10 times including the deletion of exon 1 (52 reports), the c.72+1A>G and c.137G>A variants (39 reports each) and the c.166_170del and the c.689G>A variants (21 reports each), thereby revealing several likely mutational hot spots or founder mutations.^21 24 25^ The founder Dutch mutations c.423+1G>A and *SDHB* exon 3 deletion were reported 18 and 9 times, respectively.^26^ Twenty-five per cent of the variants reported here had not been previously described.

Until recently, laboratories largely used their own methods to assess variant pathogenicity, leading to discrepancies between interpretations.^27^ Guidelines for variant interpretation were established in 2015 by the ACMG, encouraging harmonisation in variant classification.^14^ These guidelines have now been widely adopted by laboratories internationally. However, some ACMG criteria may be applied or weighted differently depending on the laboratory.^28^ Moreover, as ACMG guidelines aim at being applicable to all genes that cause Mendelian disorders, they cannot take into account the specificities of one particular gene or disease.^14^ This consideration has prompted to the development of many national or international consortia of experts on particular genes or diseases, and has led to the development of dedicated recommendations for variant interpretation. Regarding cancer predisposing genes, these international efforts have mainly been initiated for common cancers such as breast cancer or Lynch syndrome^29–31^ or ‘iconic’ tumour suppressor genes such as *TP53*.^32^ Regarding PPGL predisposition, the international NGSnPPGL study group recently published guidelines designed specifically for PPGL susceptibility genes.^2^ One of the main goals of these guidelines is to minimise the number of VUS because inconclusive report on these variants may cause confusion and anxiety for both patients and physicians. Moreover, this situation can introduce ambiguities in patient care because no established medical management for VUS carriers exists. Finally, a VUS cannot be used in predictive gene testing in relatives. Resolving the pathogenicity of a VUS is therefore essential. In the context of predisposition to PPGL, patients carrying a germline likely pathogenic or pathogenic variant in a PPGL susceptibility gene should undergo lifelong follow-up including, in the case of *SDHB*, careful monitoring for risk of metastases, GIST or RCC development.^33^ Moreover, as *SDHB*-related PPGL can occur early in childhood, predictive genetic testing and subsequent first screening by imaging, together with long-life surveillance, should be started at age 5–6 years in carriers of likely pathogenic/pathogenic SDHB variants.^34^

The ACMG and NGSnPPGL criteria were applied to assess all variants collected. The class assigned by both methods was the same in the majority of cases (n=161, 72%). In case of discordance (n=62, 28%), the ACMG classification generally assigned VUS (n=45) while the NGSnPPGL framework only assigned two VUS. These results suggest that the NGSnPPGL disease-specific recommendations are more pertinent for PPGL genetic counselling than standard ACMG criteria. In total, one or both methods ended in a VUS status for almost half of variants (n=108, 48%). After curation by experts, most were reassessed and the number of VUS decreased dramatically to 51 in total (22%). This result highlights both the challenge and the important impact of *SDHB* variant interpretation performed by experts.

In total, 77% (n=172) of variants described in the present study could be classified either as benign/likely benign (10%, n=23) or as likely pathogenic/pathogenic (67%, n=149). The relatively low rate of non-pathogenic variants is explained because frequent or well-known polymorphisms were not collected. Results of additional/functional data were helpful for classification of 94 (42%) variants. Depending on variants, we compiled results obtained from mutated tumour tissue and comprising SDHB±SDHA immunohistochemistry (n=75), LOH detection (n=26), transcriptomic clustering (n=24), cDNA analysis (n=15), western blot analysis (n=7) and measurement of SDH enzymatic activity (n=6) (online supplemental table 2 and figure 1).

Considering the whole cohort of 737 affected patients, 614 of them (83.3%) carry a likely pathogenic or a pathogenic variant. The comparison of the clinical data available for both populations (table 1) revealed no significant difference, confirming that the patient’s phenotype does not allow to classify a variant as pathogenic or not. Noticeably, the occurrence of GIST or RCC is not associated with a specific genotype as all variants identified in patients with GIST or RCC were also found in patients with PPGL only.

Finally, class 3 includes 51 variants with limited data or/and no relevant or contradictory evidence for pathogenicity such as immunohistochemistry results, mostly because no tumour tissue was available or because discordant results were obtained. As expected, VUS exclusively comprise non-truncating variants, including 37/98 missense, 10/18 non-coding variants and 3/4 in-frame indels. Moreover, we were unable to determine the pathogenicity of whole gene *SDHB* duplication.

Finally, the consensus classification (as well as pertinent information and the following comment: ‘*variant classified by experts from the NGSnPPGL study group (ENS@T/PRESSOR*)’) for the 223 collected variants has been deposited in the LOVD-SDHB database in order to share a free, valuable and updated resource with the community. International efforts should continue, and laboratories/centres are encouraged to systematically submit and review data for new variants and for already reported VUS, because reassessment may result in changes in variant classification as new evidence becomes available.

In summary, we collected *SDHB* variants worldwide and gathered an international group of curators committed to working together to expertly classify these variants. These experts undertook a joint effort to collect, share, integrate and then discuss multiple strands of evidence pertaining to *SDHB* genetic variants, with the objective of determining the clinical relevance of a variant to ultimately improve the clinical utility of genetic testing in patients and their relatives.

## Data Availability

Data are available in a public, open access repository. All data relevant to the study are included in the article or uploaded as supplementary information. Consensus classification of SDHB variants are freely available in the public Leiden Open Variation Database (LOVD) system https://databases.lovd.nl/shared/genes/SDHB.
