# Supplementary material for: International initiative for a curated SDHB variant database improving the diagnosis of hereditary paraganglioma and pheochromocytoma
Source: J Med Genet. 2021 Aug 27;59(8):785–92. doi: 10.1136/jmedgenet-2020-107652 (PMC8882202; doi:10.1136/jmedgenet-2020-107652)
Supplement: Supplementary data [file jmedgenet-2020-107652supp003.pdf]

	Benign		Pathogenic			
	**Strong**	**Supporting**	**Supporting**	**Moderate**	**Strong**	**Very Strong**
**Population data**	>1% in population databases (gnomAD, ESP)[1]			Absent in population databases (gnomAD, ESP)	Absent in population databases and increased prevalence in affecteds	
**Computational and Predictive data (*in silico* analysis)**		Multiple lines of computational evidences suggesting no deleterious impact on gene/gene product[2]<br>- missense with non predicted impact on gene/gene product<br>- silent or splicesite or intronic variant with non predicted splice impact	Multiple lines of computational evidences supporting a deleterious impact on gene/gene product[2]<br>- missense with predicted impact on gene/gene product<br>- silent or splicesite or intronic variant with predicted splice impact	Novel missense change at an amino acid residue where a different pathogenic missense change has been seen *(i.e. c.206G>T, p.Gly69Val known as pathogenic and novel missense c.206G>A, p.Gly69Asp)*	Same amino acid change as an established pathogenic variant *(i.e. c.39G>T, p.Leu13Phe known as pathogenic and novel missense c.39G>C, p.Leu13Phe)*	Null variants[3]: nonsense, frameshift, canonical splice site, initiation codon, single exon or multi-exon deletion/duplication with open-reading frame disruption
**Additional data**	In tumor DNA, loss of the allele carrying the variant	1) Immunohistochemistry evidence: positive SDHB staining<br>2) No loss of heterozygosity<br>3) cDNA analysis showing no unexpected exon size nor exon skipping (for likely splicing variants)<br>4) Transcriptomic analysis: tumor classification not in *SDHx*-related cluster (C1A)	Loss of heterozygosity in tumor DNA	1) Immunohistochemistry evidence: negative SDHB staining but without genotyping[4] of all *SDHx* genes (*SDHA, SDHB, SDHC, SDHD*)<br>2) SDH enzymatic activity abolished without genotyping[4] of all *SDHx* genes (*SDHA, SDHB, SDHC, SDHD*)<br>3) Western blot evidence: no SDHB protein detected but without genotyping[4] of all *SDHx* genes (*SDHA, SDHB, SDHC, SDHD*)<br>4) Transcriptomic analysis: tumor classification in C1A cluster but without genotyping[4] of all *SDHx* genes (*SDHA, SDHB, SDHC, SDHD*)	1) Immunohistochemistry evidence: SDHB negative and SDHA positive staining with *SDHB, SDHC* and *SDHD* sequencing but without search for *SDHx* large deletions or duplications<br>2) cDNA analysis : abnormal splicing<br>3) SDH enzymatic activity abolished with *SDHx* genes sequencing but without search for *SDHx* large deletions or duplications<br>4) Western blot evidence: no SDHB protein detected with *SDHx* genes sequencing but without search for *SDHx* large deletions or duplications<br>5) Transcriptomic analysis: tumor classification in C1A cluster with *SDHx* genes sequencing but without search for *SDHx* large deletions or duplications	1) Immunohistochemistry evidence:<br>- negative SDHB staining with genotyping[4] of all *SDHx* genes (*SDHA, SDHB, SDHC, SDHD*)<br>- negative SDHB and positive SDHA staining with genotyping[4] of *SDHB, SDHC* and *SDHD* genes<br>2) SDH enzymatic activity abolished with genotyping[4] of all *SDHx* genes (*SDHA, SDHB, SDHC, SDHD*)<br>3) Western blot evidence: no SDHB protein detected with genotyping[4] of all *SDHx* genes (*SDHA, SDHB, SDHC, SDHD*)<br>4) Transcriptomic analysis: tumor classification in C1A cluster with genotyping[4] of all *SDHx* genes (*SDHA, SDHB, SDHC, SDHD*)
**Segregation data**	Non-segregation with disease		Co-segregation with disease in multiple affected family members	Increased segregation data →		
**Allelic data**		Observed in *cis/trans* with a pathogenic variant				
**Publication data[5]**	← Increased publication data	Published as benign variant	Published as pathogenic variant	Increased publication data →		

[1] Stand-alone evidence of benign impact if >5% in population databases

[2]Combination of different *in silico* tools (missense: PolyPhen2, SIFT, MutationTaster; splice site: MaxEntScan, NNSplice) and Conservation data (nucleotide: phyloP; amino acid: Orthologs conservation, distance between amino acids: Grantham distance)

[3]Except variant occuring in the last exon or in tle last 50 bps of the penultimate exon. Such variants cannot be interpreted without additional assay

[4]« Genotyping » includes both sequencing using NGS or Sanger and search for large duplication or deletion and implies that no additional *SDHx* variant was found

[5]Well-documented publications (number of cases published, functional data…)

**Variant classification**	**1rst criterion**	**2nd criterion**
Pathogenic	1 Very Strong	≥1 Strong
		≥2 Moderate
		1 Moderate + 1 Supporting
		≥2 Supporting
	≥2 Strong	-
	1 Strong	≥3 Moderate
		2 Moderate + ≥2 Supporting
		1 Moderate + ≥4 Supporting
Likely Pathogenic	1 Very Strong	1 Moderate
	1 Strong	1-2 Moderate
	1 Strong	≥2 Supporting
	≥3 Moderate	-
	2 Moderate	≥2 Supporting
	1 Moderate	≥4 Supporting
Likely Benign	1 Strong	1 Supporting
	≥2 Supporting	-
Benign	1 Stand-Alone	-
	≥2 Strong	-

Supplemental Figure 1: adjusted ACMG criteria and combination used for SDHB variant classification
