# Supplementary material for: International initiative for a curated SDHB variant database improving the diagnosis of hereditary paraganglioma and pheochromocytoma
Source: J Med Genet. 2021 Aug 27;59(8):785–92. doi: 10.1136/jmedgenet-2020-107652 (PMC8882202; doi:10.1136/jmedgenet-2020-107652)
Supplement: Supplementary data [file jmedgenet-2020-107652supp004.pdf]

Variant classification according to the NGSnPPGL recommendations	
Pathogenic	Variant reported in literature with strong evidence of pathogenicity<br>or<br>Null variant with functional evidence for pathogenicity
Likely Pathogenic	Null variant with no material available for functional study<br>or<br>Missense variant with ≥3 *in silico* predictions in favour of pathogenicity and functional study supportive of a damaging effect<br>or<br>Intronic or silent variant with predicted splice impact by *in silico* analysis and and functional study supportive of a damaging effect
VUS	Insufficient evidence to classify<br>or<br>Contradictory criteria
Likely Benign	Missense variant with ≥3 in silico predictions in favour of the variant being benign<br>or<br>Intronic or silent variant with no predicted splice impact<br>or<br>Co-occurrence with pathogenic variant<br>or<br>Functional evidence for non-pathogenicity
Benign	AF>1% in control groups<br>or<br>Presence in control groups with no co-segregation with the disease<br>or<br>AF=0,01-1% and functional evidence for non-pathogenicity

Supplemental Figure 2: Variant classification according to the NGSnPPGL recommendations
